# Supplementary figures and images for: KLHDC3 deficiency in mice reveals essential roles in development, survival, and adiposity via the DesCEND ubiquitin pathway
Source: BMC Genomics. 2026 Jan 28;27:222. doi: 10.1186/s12864-026-12574-5 (PMC12922316; doi:10.1186/s12864-026-12574-5)

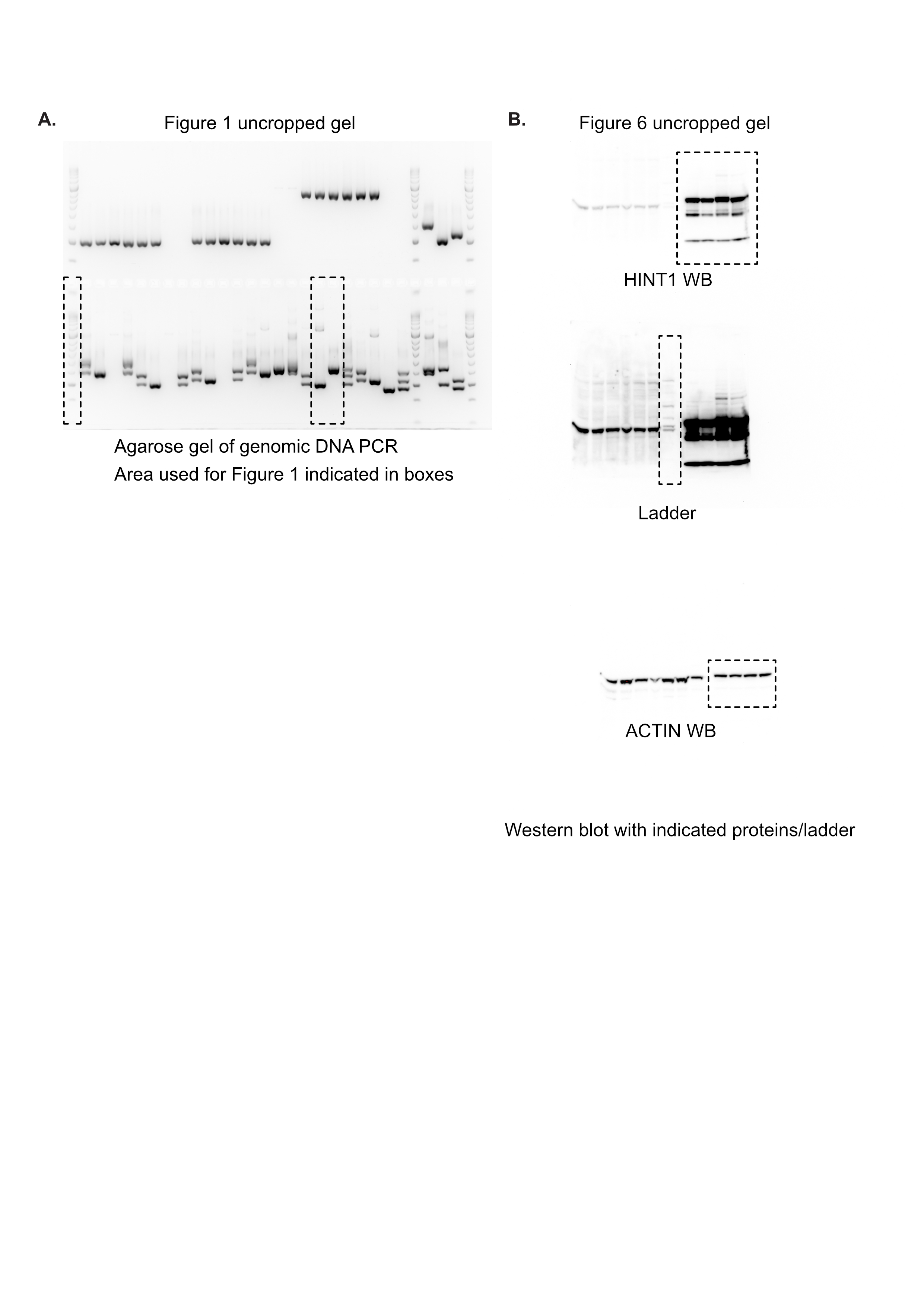

Supplement: Supplementary file 1 — Supplementary Material 1. Supplemental Figure S1 – Uncropped gel images (A) Uncropped gel image from Panel 1E. Agarose gel of genomic DNA PCR for genotyping. Bands used in main figure indicated in the boxed areas. (B) Uncropped gel image from Panel 6D. Western blot; bands used in main figure indicated in the boxed areas [file 12864_2026_12574_MOESM1_ESM.jpg]
