# Supplementary material for: KLHDC3 deficiency in mice reveals essential roles in development, survival, and adiposity via the DesCEND ubiquitin pathway
Source: BMC Genomics. 2026 Jan 28;27:222. doi: 10.1186/s12864-026-12574-5 (PMC12922316; doi:10.1186/s12864-026-12574-5)
Supplement: Supplementary file 2 — Supplementary Material 2. Supplemental Datasets [file 12864_2026_12574_MOESM2_ESM.zip › supp.docx]

**Supplemental Information**

**Supplemental Datasets**

| **Name** | **Description** | **GEO accession number** |
| --- | --- | --- |
| **Supplemental Dataset 1** | Full histopathology reports of 2 *Klhdc3^+/+^* and 2 *Klhdc3^-/-^* animals (1 male and 1 female per genotype) | Not applicable |
| **Supplemental Dataset 2** | RNA-seq from mouse embryonic fibroblasts (MEFs) | GSE271979 |
| **Supplemental Dataset 3** | RNA-seq from immortalised myeloid cell lines | GSE271980 |
| **Supplemental Dataset 4** | Proteomic analysis of MEFs | Not applicable |
