# Supplementary material for: KLHDC3 deficiency in mice reveals essential roles in development, survival, and adiposity via the DesCEND ubiquitin pathway
Source: BMC Genomics. 2026 Jan 28;27:222. doi: 10.1186/s12864-026-12574-5 (PMC12922316; doi:10.1186/s12864-026-12574-5)
Supplement: Supplementary file 2 — Supplementary Material 2. Supplemental Datasets [file 12864_2026_12574_MOESM2_ESM.zip › Supp-Dataset-1_Histopathology report.pdf]

Contacts:

Phenomics Australia Histopathology and
Slide Scanning Service
The University of Melbourne
Department of Anatomy and Physiology
Grattan Street, PARKVILLE, VIC 3010

9.1 Histopathology Report

Case Number APN23/043 St Vincent's Institute of Medical Research (Carl Walkley)

Registration Date Thu 17/08/2023

Animal Details
124, WT +/+
DOB: 16/06/23, 8 weeks, Male, 24.4g, Black
Strain: Klhdc3

129, WT +/+
DOB: 16/06/23, 8 weeks, Female, 18.8g, Black
Strain: Klhdc3

125, KO -/-
DOB: 16/06/23, 8 weeks, Male, 17.7g, Black
Strain: Klhdc3

128, KO -/-
DOB: 16/06/23, 8 weeks, Female, 16.2g, Black
Strain: Klhdc3

DoD / Necropsy Wed 23/08/2023

Death CO2

Origin St Vincent's Institute of Medical Research

Treatment
Klhdc3-/-
General histopathology assessment; ? the Klhdc3-/- are infertile (small number attempted to breed but unsuccessful so far)
C. Walkley 6th July 2023

Species / Breed / Strain C57BL/6

Animal Health Facility
St. Vincent's Institute Bioresources Centre
POSITIVE for:
Helicobacter spp
Chilomastix bettencourti
Entamoeba muris
Tritrichomonas muris
Mouse Norovirus

Organs Examined Adrenal glands, Bladder, Bone marrow, Brain, Cecum, Cervix, Clitoral gland, Colon, Duodenum, Epididymes, Eyes, Gall bladder, Harderian glands, Head, Heart, Hind leg (Long bone, Bone marrow, Synovial joint, Skeletal muscle), Ileum, Jejunum, Kidney, Liver, Lungs, Mammary tissue, Mesenteric lymph node, Sternum, Ovaries, Oviducts, Pancreas, Penis, Preputial gland, Prostate glands, Salivary glands and Regional lymph nodes, Seminal vesicles, Skin, Spinal cord, Spleen, Stomach, Tail, Testes, Thymus, Thyroids, Trachea, Uterus, Vagina

Macroscopic Observations
P.A. iLab request number: PAHSSS-CW-410
Date of Necropsy confirmed by P.A staff: Necropsy 23/08/2023

Courier/Transportation details: JetPets job #1525297

Animal details:
Klhdc3 mutants
124, WT +/+ Male
129, WT +/+ Female
125, KO -/- Male
128, KO -/- Female

Body Condition Scoring (BCS): Scale of 1-5.
5: The mouse is obese, and bones cannot be felt at all
4: The mouse is well-fleshed, and bones are barely felt
3: The mouse is in optimal condition. The bones are palpable but not prominent
2: The mouse is thin, and bones are prominent
1: Muscle wasting is advanced; fat deposits are gone, and bones are very prominent.

All animals in this cohort (mutants & controls) scored a BCS of 3

At the time of necropsy, the animals appeared well nourished, well groomed, active/curious (rearing on hind legs) and healthy with normal movement and gait. There were no observable dermal lesions, typical oral features, unremarkable dentition and no nasal/ocular discharges. The coat appeared well groomed and smooth. No observable abnormalities of the limbs, paws and digits. The gastrointestinal tract contained ample ingesta and the thoracic and abdominal viscera showed no macroscopic abnormalities. Please refer to individual animals for more macroscopic details.
Animal 128, KO -/- Female -Uterus was observed to be thin and delicate. The ovaries were inconspicuous.

Blood Report:
Blood results show comparable results between the mutant animals and wild type controls in this cohort.
Animals 124, WT +/+ Male, 129, WT +/+ Female and 125, KO -/- Male show a mildly elevated haematocrit (HCT) reading. This is often due to mild dehydration when the red blood cell (RBC) count and mean cell volumes (MCV) are within normal range.
124, WT +/+ Male, 125, KO -/- Male and 128, KO -/- Female animals show a white blood cell count (WBC) outside the normal reference interval (elevated and low).
For more details please see the accompanying APN23/043SVI(C.Walkley) Blood Report.

Notes:
(1) Haematology values can vary with mouse strain/stock, age, sex, blood sampling method, fasting and environmental conditions, pathogen status, and laboratory.
(2) Decreased platelet counts are a common haematologic finding in mice. This change is often secondary to blood collection difficulties rather than a true decrease in platelet counts. Mouse platelets readily aggregate and instrument generated platelet counts will underestimate true platelet counts in the presence of platelet clumping. In addition, mouse platelet clumps are counted as eosinophils by some automated haematology analysers. A high number of large platelets (high MPV) with a low platelet count suggests the bone marrow is producing platelets and releasing them into circulation rapidly.

The reference intervals used for this report are based on published values of adult mice at 8 weeks old.

REFERENCES:
(1) National Toxicology Program-Nonneoplastic Lesion Atlas
https://ntp.niehs.nih.gov
(2) Maronpot, R.R (1999) Pathology of the Mouse, USA: Cache River Press

NOTES
(1)Compared to other mammalian species, the mouse red blood cell has a short lifespan compared to other species. Red blood cell lifespan has been estimated at between 30 and 40 days (Bannerman, 1983; Car and Eng, 2001). In contrast, the red blood cell lifespan of other species is much longer (rats 45-50 days, dogs 110 days, and humans 120 days). Because the lifespan of murine red cells is so short, there are a higher percentage of circulating immature red blood cells at any given time. Immature red blood cells are larger than mature erythrocytes and stain with a blue tint. Therefore, polychromasia (blue-staining cells) and anisocytosis (variably sized red blood cells) occur to a greater extent in normal healthy mice, compared to humans and many other animals.
Reference: Hematology of the Mouse, Nancy Everds Haskell Laboratory for Health and Environmental Sciences, Newark, Delaware, USA

Microscopic Observations

Summary
124, WT +/+ Male
No lesions of significance
Neuropathology to comment on CNS

129, WT +/+ Female
Uterus - Large numbers of neutrophils are present within the vaginal lumen. The micromorphology of the uterus and vagina places the animal at metestrus (96758)
Neuropathology to comment on CNS

125, KO -/- Male
Peripheral blood smear- Conspicuous polychromasia, a common feature in murine haematopathology (96705)
Heart- Query vacuolation/degeneration (96778)
Kidney- Proteinaceous material in Bowman's space. Occasional mild perivascular lymphocyte aggregates (96717)
Skin-standard ventral- Mild focus of lymphocytic infiltrates in the dermis (96719)
Reproductive organs- Testes-No lesions of significance (96707)
Neuropathology to comment on CNS

128, KO -/- Female
Mammary glands -Paucity of lactiferous ducts when compared to 129 wild type control (96743)
Uterus - the endometrium is discernibly thinner when compared to 129 wild type female uterus (96732-96735)
Ovaries/oviducts-No lesions of significance
Neuropathology to comment on CNS

124 (control)

Macro Observations

Tail suspension test for neurological defects – negative.
Dentition, tongue and oral cavity was unremarkable.
BCS: 3
Testes: 7x4x4mm, symmetrical
Spleen: 14x4x2mm
Kidneys: 11x5x4mm, symmetrical
Thymus: 6x6x2mm

Lungs inflated.
Heart: 9x5x4mm
Brain: 15x10x5mm, symmetrical
Pituitary gland identified, macroscopically normal
Tail length: 80mm (straight)
Head harvested for evaluation of auditory and vestibular structures.
Bone marrow smear taken from left hind leg.

No macroscopic lesions identified.

Micro Observations

Animal 124 was used as a male histological control.

Marrow smear: Examination of the smear shows representative cells from the myeloid and lymphoid series. There doesn't appear to be a predominance in any cell type (96702).

Peripheral blood smear: Examination of the smear shows red blood cells (majority of cells shown), a number of monocytes, platelets/ clumps and segmented neutrophils. Scant polychromasia.
No discernible morphological changes or detectable parasites (96703)

Organs examined: Testes/Epididymes (96678, 96679), Seminal vesicles (96680, 96681), Prostate glands (96680, 96681), Penis/Preputial gland (96682), Urinary Bladder (96680, 96681), Liver/Gall bladder (96683), Stomach (96684), Duodenum/jejunum/ileum/GALT (96684, 96685, 96686, 96779), Cecum/Colon/GALT (96686), Mesenteric Lymph Node (96687, 96688), Spleen (96680, 96681), Pancreas (96680, 96681), Kidneys (96689), Salivary Glands/regional lymph nodes (96687, 96688), Thyroid glands/Parathyroid glands (96690), Trachea/Lungs (96690), Thymus (96690), Heart (96690), Skin-ventral (96691), Tail (96692), Eyes/Harderian Glands (96693), Brain (96694), Spinal cord (96695, 96696), Hind leg (96697, 96698), Head (96699, 96700), Sternum (96701).

Summary:
No lesions of significance
Neuropathology to comment on CNS

129 (control)

Macro Observations

Tail suspension test for neurological defects – negative.
Dentition, tongue and oral cavity was unremarkable.
BCS: 3
Spleen: 14x4x2mm
Kidneys: 10x5x4mm, symmetrical
Thymus: 7x6x2mm
Lungs inflated.
Heart: 9x5x4mm
Brain: 14x5x4mm, symmetrical
Pituitary gland identified, macroscopically normal
Tail length: 78mm (straight)
Head harvested for evaluation of auditory and vestibular structures.
Bone marrow smear taken from left hind leg.

No macroscopic lesions identified.

Micro Observations

Animal 129 was used as a female histological control.

Marrow smear: Examination of the smear shows representative cells from the myeloid and lymphoid series. There doesn't appear to be a predominance in any cell type (96755).

Peripheral blood smear: Examination of the smear shows red blood cells (majority of cells shown), a number of monocytes, platelets/clumps and segmented neutrophils. Scant polychromasia.
No discernible morphological changes or detectable parasites (96756)

Organs examined: Mammary glands (96767), Ovaries/oviducts (96757, 96758), Uterus/cervix/vagina (96757, 96758), Urinary Bladder (96757, 96758), Liver (96759), Stomach (96760), Duodenum/jejunum/ileum/GALT (96761), Cecum/Colon/GALT (96762), Mesenteric Lymph Node (96763, 96764), Spleen (96757, 96758), Pancreas (96757, 96758), Kidneys/Adrenal glands (96765), Salivary Glands/regional lymph nodes/cervical mammary glands (96763, 96764), Thyroid/Parathyroid glands (96766), Trachea/Lungs (96766), Thymus (96766), Heart (96766), Skin-standard ventral (96767), Tail (96768), Eyes/Harderian Glands (96769), Brain (96770), Spinal cord (96771, 96772), Hind leg (96773, 96774), Head (96775, 96776), Sternum (96777).

Summary:
No lesions of significance
Uterus (96758, 96757)- Large numbers of neutrophils are present within the vaginal lumen. The micromorphology of the uterus and vagina places the animal at metestrus.

Neuropathology to comment on CNS

125

Macro Observations

Tail suspension test for neurological defects – negative.
Dentition, tongue and oral cavity was unremarkable.
BCS: 3
Testes: 7x5x4mm, symmetrical
Spleen: 18x5x2mm
Kidneys: 12x7x6mm, symmetrical
Thymus: 7x7x2mm
Lungs inflated.
Heart: 12x6x5mm
Brain: 16x10x5mm, symmetrical
Pituitary gland identified, macroscopically normal
Tail length: 75mm (straight)
Head harvested for evaluation of auditory and vestibular structures.
Bone marrow smear taken from left hind leg.

Animal was considered small, query age related.

No macroscopic lesions identified.

Micro Observations

Marrow smear: Examination of the smear shows representative cells largely from the myeloid series. There doesn't appear to be a predominance in any cell type (96704).

Peripheral blood smear: Examination of the smear shows red blood cells (majority of cells shown), a number of monocytes, platelet cells/clumps and segmented neutrophils. Conspicuous polychromasia, a common feature in murine haematopathology. Polychromasia is not as conspicuous in the male and female wild type control smears.
No discernible morphological changes or detectable parasites (96705)

Summary:
Peripheral blood smear- Conspicuous polychromasia, a common feature in murine

haematopathology (96705)
Heart- Query vacuolation/degeneration (96778)
Kidney- Proteinaceous material in Bowman's space. Occasional mild perivascular lymphocyte aggregates (96717)
Skin-standard ventral- Mild focus of lymphocytic infiltrates in the dermis (96719)

Neuropathology to comment on CNS

Testes/Epididymes

Section shows typical convoluted seminiferous tubules at various stages of cycle surrounded by the tunica albuginea. Within the tubules, unremarkable spermatogenic cells including, Sertoli cells, spermatogonia, developing spermatocytes and spermatids. Typical interstitial Leydig cells.
Section also shows unremarkable vas deferens with typical intraluminal sperm.
The architecture of the epididymis is typical, with numerous intraluminal elongated spermatozoa.
No lesions of significance
(96706, 96707)

Seminal vesicles

Unremarkable tall columnar epithelium and folded mucosa.
Presence of typical intraluminal eosinophilic secretions
No lesions of significance
(96708, 96709)

Prostate glands

Section shows unremarkable dorsal lateral ventral and coagulating glands with typical intraluminal
secretions. Section also includes unremarkable urethra and proximal vas deferens.
No lesions of significance
(96708, 96709)

Penis/Preputial gland

Section shows typical penile structures including prepuce, glans, corpus cavernosum and urethra. Typical dilated preputial glands including basal and secretory cells. Section also includes bulbourethral glands (Cowper’s glands) located near the base of the penis.
No lesions of significance
(96710)

Urinary Bladder

Unremarkable distended bladder with typical urothelium and detrusor muscle.
No lesions of significance
(96708, 96709)

Liver/Gall bladder

Section shows typical liver parenchyma including hepatocytes, Kupffer cells, portal triads and central veins. Unremarkable extra- hepatic bile ducts.
No lesions of significance
(96711)

Stomach

Section shows unremarkable fore and glandular portions of the stomach with limiting ridge.
Section also includes pyloric sphincter and duodenal bulb with Brunner's glands.
No lesions of significance
(96712)

Small Intestine (Duodenum, Jejunum & Ileum)/GALT

Section shows typical mucosal villi and submucosal layers. Distended gut with abundant intraluminal content. Peyer's patches display typical reactive nodal histology.
No lesions of significance
(96713)

Cecum/Colon/GALT

Typical mucosal folds and submucosal layers. Well formed intraluminal content. Unremarkable muscularis and ganglion cells of the plexuses. Unremarkable cryptopatch.
No lesions of significance
(96714)

Mesenteric lymph node

Mesenteric lymph node with typical nodal histology.
No lesions of significance
(96715, 96716)

Spleen

Unremarkable follicular pattern identified with typical red and white pulp micromorphology.
No lesions of significance
(96708, 96709)

Pancreas

Section shows representative exocrine tissue (serous acini) and endocrine tissue (islets of Langerhans).
No lesions of significance
(96708, 96709)

Kidney

Section shows representative cortex, medulla, and papilla regions. Largely typical glomeruli, many with open capillary lumens. In a number of Glomeruli, there is proteinaceous material in Bowman's space. The interstitium and tubules are unremarkable. Occasional mild perivascular lymphocyte aggregates. Section includes renal lymph node with typical nodal histology.
(96717)

Comments:

Pathology to comment

Adrenal glands

Section shows adrenal glands with typical cortex/medulla micromorphology.
No lesions of significance
(96717)

Salivary glands and Regional lymph nodes

Section shows unremarkable submandibular, sublingual and parotid glands. Section also includes unremarkable lacrimal gland. Regional lymph nodes with typical nodal histology.
No lesions of significance
(96715, 96716)

Thyroids

Normal thyroid glands with typical colloid secreting follicles lined by cuboidal epithelium.
No lesions of significance
(96718, 96778)

Trachea/Lungs

Section shows lung parenchyma/alveoli, bronchioles/airways, vasculature and peripheral lymph nodes.
Small portion of trachea with unremarkable mucosal epithelial lining and hyaline cartilage.
Oesophagus with typical features including stratified squamous epithelium.
No lesions of significance
(96718, 96778)

Thymus

Section shows typical medulla/cortex distribution and micromorphology.
No lesions of significance
(96718, 96778)

Heart/chambers/vessels/valves

Typical micromorphology observed in cardiac muscle, chambers, valves and great vessels of the heart. The cardiac muscle fibres demonstrate typical features including central nuclei, branching fibres and striations.
No lesions of significance
(96718, 96778)

Comments:

Pathology to comment
Query vacuolation/degeneration

Skin

Typical dermal appendages and distribution. Mild focus of lymphocytic infiltrates in the dermis.
Unremarkable thin layer of striated muscle (panniculus carnosus) and subjacent abdominal muscle.
(96719)

Comments:

Pathology to comment

Tail

Section shows typical tail components including keratinized squamous epithelium, dense regular connective tissue, tendons, caudal vertebra, bone marrow/adipocytes, intervertebral disc, skeletal muscle,
nerves and blood vessels.
No lesions of significance
(96720)

Eyes/Harderian glands

Section shows eyes with unremarkable retina (pigmented), cornea, iris, ciliary body, lens, sclera and choroid.
Typical branched tubuloalveolar formation of the Harderian gland.
Section also includes portion of unremarkable optic nerve and extraocular muscles.
No lesions of significance
(96721)

Brain

Sections were prepared from the standard levels of the brain:

Level I: including cortex, corpus callosum, caudate putamen, lateral ventricles (approx. Bregma 0.98mm)

Level II: including the hippocampus, thalamus, hypothalamus and lateral and third and lateral ventricles (approx. Bregma -1.82mm)

Level III: includes the cerebellum, pons and fourth ventricle (approx. Bregma -5.68mm)

Sections of brain appear symmetrical with unremarkable meninges and typical lamination. The neocortical layers appear to align with the wild type control brains.
The cerebellum appears symmetrical with typical architecture and Purkinje cells.
There is no obvious neuronal loss and the myelination appears normal.
(96722)

Comments:

Neuropathology to comment

Spinal cord

Representative thoracic, lumbar and sacral region of spinal cord, vertebral bone, intervertebral disc, striated muscle, peripheral nerves, brown adipose tissue, and bone marrow.
No lesions of significance
(96723, 96724)

Comments:

Neuropathology to comment

(Hind leg) Long bone/Bone marrow/Synovial joint/Skeletal muscle

Section shows unremarkable long bone, bone marrow, striated muscle, synovial joint, tarsal bones, digit bones, cornified foot pad with discernible eccrine glands and representative nail bed/nail. Also a number of representative nerve bundles and tendons are discernible.
The skeletal muscle shows consistent fibre size with peripheral nuclei.
No lesions of significance
(96725, 96726)

Head

Multiple levels through the head demonstrate dermal appendages, nasal cavity with unremarkable nasal epithelium, oral cavity, dentition, and tongue with keratinized papillae.
Sections also show unremarkable pituitary gland including pars intermedia, pars distalis and pars nervosa as well as the trigeminal nerve/ganglia (96727).
The outer and middle regions of the ear are discernible. The tympanic membrane is intact, and the ossicles are unremarkable and include the stapedial annular ligaments.
Typical components of the inner ear including bony labyrinth, organ of corti, stria vascularis and scala cavities are discernible. The organ of corti is unremarkable with no discernible loss of outer hair cells and typical tectorial membrane.
The cochlear nerve and spiral ganglion is also demonstrated and based on representative levels, there is no reduction in the density of the spiral ganglion cells. Examples of otolith organs can be seen with typical features such as the hair cells and mineral otoliths.
No lesions of significance
(96727, 96728)

Other organ / tissue

Sternum: Section shows representative sternebrae, costal cartilage, intersternebral joint, intercostal skeletal muscle and brown fat.
Section shows haematopoietic tissue islands and vascular sinuses interspersed within a meshwork of trabecular bone. The bone marrow morphology demonstrates typical myeloid cells including megakaryoblasts and lymphoid features. There doesn't appear to be a predominance in any cell type.
No lesions of significance
(96729)

128

Macro Observations

Tail suspension test for neurological defects – negative.
Dentition, tongue and oral cavity was unremarkable.
BCS: 3
Spleen: 15x5x2mm
Kidneys: 10x8x6mm, symmetrical
Thymus: 7x7x2mm
Lungs inflated.
Heart: 10x8x6mm
Brain: 16x11x6mm, symmetrical
Pituitary gland identified, macroscopically normal
Tail length: 75mm (straight)
Head harvested for evaluation of auditory and vestibular structures.
Bone marrow smear taken from left hind leg.

Delicate/thin uterus. Inconspicuous ovaries.
Animal was considered small, query age related.

No macroscopic lesions identified.

Micro Observations

Marrow smear: Examination of the smear shows representative cells from the myeloid and lymphoid series. There doesn't appear to be a predominance in any cell type (96730).

Peripheral blood smear: Examination of the smear shows red blood cells (majority of cells shown), a number of monocytes, platelet clumps and segmented neutrophils. Discernible polychromasia but not as florid as mutant male 125.
No discernible morphological changes or detectable parasites (96731)

Summary:
Mammary glands (96743)-Paucity of lactiferous ducts when compared to 129 wild type control.
Uterus (96732-96735)- the endometrium is discernibly thinner when compared to 129 wild type female uterus.

Neuropathology to comment on CNS

Mammary glands

Section shows mammary fat pad with a few lactiferous ducts, a large lymph node with typical nodal histology, white adipose tissue and subjacent abdominal muscle. The number and size of the lactiferous ducts are noticeably fewer and smaller compared to 129 wild type control mammary glands.
(96743)

Comments:

Pathology to comment

Ovaries/Oviducts

Multiple levels show bilateral ovaries containing follicles at various stages of development (primary through to antral) and a corpus luteum. Unremarkable oviduct micromorphology with typical columnar epithelium and mucosal folds.
No lesions of significance
(96732-96735)

Uterus/Cervix/Vagina/Clitoral gland

Uterus comprising of lateral horns. Discernible endometrium/endometrial glands, myometrium, and adventitia. The endometrium is thinner with fewer glands when compared to 129 wild type female uterus. Worth noting that stage of estrus influences the appearance and thickness of the uterus.
(96732-96735)

Comments:

Pathology to comment

Urinary Bladder

Unremarkable collapsed bladder with typical urothelium and detrusor muscle.
No lesions of significance
(96732-96735)

Liver/Gall bladder

Section shows typical liver parenchyma including hepatocytes, Kupffer cells, portal triads and central veins.
Unremarkable Gall bladder.
No lesions of significance
(96736)

Stomach

Section shows unremarkable fore and glandular portions of the stomach with limiting ridge.
Some mechanical artefact affecting the non-glandular stomach.
Section also includes pyloric sphincter and duodenal bulb with Brunner's glands.
No lesions of significance
(96737)

Small Intestine (Duodenum, Jejunum & Ileum)/GALT

Section shows typical mucosal villi and submucosal layers. The gut lumen is distended with abundant loose intraluminal content. Peyer’s patch and mesenteric lymph node display typical nodal histology.
No lesions of significance
(96738)

Cecum/Colon/GALT

Typical mucosal folds and submucosal layers. Well formed intraluminal content. Unremarkable muscularis and discernible ganglion cells of the plexuses and typical lymphoid clusters (cryptopatches).
No lesions of significance
(96739)

Mesenteric lymph node

Mesenteric lymph node with typical nodal histology.
No lesions of significance
(96740, 96754)

Spleen

Unremarkable follicular pattern identified with typical red and white pulp micromorphology.
No lesions of significance
(96732-96735)

Pancreas

Section shows representative exocrine tissue (serous acini) and endocrine tissue (islets of Langerhans).
No lesions of significance
(96732-96735)

Kidney

Section shows representative cortex, medulla, and papilla regions. Typical glomeruli, many with open capillary lumens. The interstitium and tubules are unremarkable. Section includes renal lymph node with typical nodal histology.
No lesions of significance
(96741)

Adrenal glands

Section shows adrenal glands with typical cortex/medulla micromorphology.
No lesions of significance
(96741)

Salivary glands and Regional lymph nodes

Section shows unremarkable submandibular, sublingual and parotid glands. Regional lymph nodes with typical nodal histology. Section includes portion of cervical mammary fat pad with no obvious lactiferous ducts.
No lesions of significance
(96740, 96754)

Thyroids

Unremarkable colloid secreting follicles identified. Section also shows unremarkable parathyroid gland.
No lesions of significance
(96742)

Trachea/Lungs

Section shows typical lung parenchyma/alveoli, bronchioles/airways, vasculature and peripheral lymph nodes.
Oesophagus with typical features including stratified squamous epithelium.
No lesions of significance
(96742)

Thymus

Section shows typical medulla/cortex distribution and micromorphology.
No lesions of significance
(96742)

Heart/chambers/vessels/valves

Typical micromorphology observed in cardiac muscle, chambers, valves and great vessels of the heart. The cardiac muscle fibres demonstrate typical features including central nuclei, branching fibres and striations.
No lesions of significance
(96742)

Skin

Typical dermal appendages and distribution. Unremarkable thin layer of striated muscle (panniculus carnosus) and subjacent abdominal muscle. Section includes mammary glands and subcutaneous lymph node.
No lesions of significance
(96743)

Tail

Section shows typical tail components including keratinized squamous epithelium, dense regular connective tissue, tendons, caudal vertebra, bone marrow/fat, intervertebral disc, skeletal muscle, nerves and blood vessels.
No lesions of significance
(96744)

Eyes/Harderian glands

Section shows eyes with unremarkable retina (pigmented), cornea, iris, ciliary body, lens, sclera and choroid.
Typical branched tubuloalveolar formation of the Harderian gland.
Section also includes portion of unremarkable optic nerve and extraocular muscles.
No lesions of significance
(96745)

Brain

Sections were prepared from the standard levels of the brain:

Level I: including cortex, corpus callosum, caudate putamen, lateral ventricles (approx. Bregma 2.10mm)

Level II: including the hippocampus, thalamus, hypothalamus and lateral and third and lateral ventricles (approx. Bregma -1.82mm)

Level III: includes the cerebellum, pons and fourth ventricle (approx. Bregma -5.80 to -5.88mm)

Sections of brain appear symmetrical with unremarkable meninges and typical lamination. The neocortical layers appear to align with the wild type control brains.
The cerebellum appears symmetrical with typical architecture and Purkinje cells.
There is no obvious neuronal loss and the myelination appears normal.
(96746)

Comments:

Neuropathology to comment

Spinal cord

Representative thoracic, lumbar region of spinal cord, vertebral bone, intervertebral disc, striated muscle, peripheral nerves, brown adipose tissue, and bone marrow.
No lesions of significance
(96747, 96748)

Comments:

Neuropathology to comment

(Hind leg) Long bone/Bone marrow/Synovial joint/Skeletal muscle

Section shows unremarkable long bone, bone marrow, striated muscle, synovial joint, tarsal bones, digit bones, cornified foot pad with discernible eccrine glands and representative nail bed/nail. Also a number of representative nerve bundles and tendons are discernible.
The skeletal muscle shows consistent fibre size with peripheral nuclei.
No lesions of significance
(96749, 96750)

Head

Multiple levels through the head demonstrate dermal appendages, nasal cavity with unremarkable nasal epithelium, oral cavity, dentition, and tongue with keratinized papillae.
Sections also show unremarkable pituitary gland including pars intermedia, pars distalis and pars nervosa as well as the trigeminal nerve/ganglia (96751).
The outer and middle regions of the ear are discernible. The tympanic membrane is intact, and the ossicles are unremarkable and include the stapedial annular ligaments.
Typical components of the inner ear including bony labyrinth, organ of corti, stria vascularis and scala cavities are discernible. The organ of corti is unremarkable with no discernible loss of outer hair cells and typical tectorial membrane.
The cochlear nerve and spiral ganglion is also demonstrated and based on representative levels, there is no reduction in the density of the spiral ganglion cells. Examples of otolith organs can be seen with typical features such as the hair cells and mineral otoliths.
No lesions of significance
(96751, 96752)

Other organ / tissue

Sternum: Section shows representative sternebrae, costal cartilage, intersternebral joint, intercostal skeletal muscle and brown fat.
Section shows haematopoietic tissue islands and vascular sinuses interspersed within a meshwork of trabecular bone. The bone marrow morphology demonstrates typical myeloid cells including megakaryoblasts and lymphoid features. There doesn't appear to be a predominance in any cell type.
No lesions of significance
(96753)

Comment / Plan

Representative slides for case APN23/043 (C. Walkley) have been referred

22nd September 2023

Supplementary Pathology Report

124 (control)

No abnormalities detected

129 (control)

96757- acute (neutrophilic) vaginitis with mild infiltration (and degeneration) of surface epithelium

125

96778- heart- focal cardiomyocyte necrosis and loss with pooling of plasma protein; surrounding myocytes sometimes show cytoplasmic hypereosinophilia and loss of striations; few Anitschov cells with “caterpillar “ nuclear chromatin

96717- kidney- small amount of protein in Bowman’s spaces but glomeruli normal by Light Microscopy.

96706, 96707- testes-No abnormalities detected

96719- skin- very mild focal dermatitis with a few aggregates of mononuclear cells and neutrophils

128

96732- 96733- 96734- 96735- uterine atrophy with marked attenuation of wall, the condensed stroma containing a few remnant glands
96743 - mammary glands - deficiency of the lactiferous ducts

Summary

Please refer to comments above

Supplementary Neuropathology Report

124 (control)

Brain-No abnormalities detected
Spinal cord - No abnormalities detected

129 (control)

Brain-No abnormalities detected
Spinal cord - No abnormalities detected

125

Brain-No abnormalities detected
Spinal cord - No abnormalities detected

128

Brain-No abnormalities detected
Spinal cord - No abnormalities detected

Summary

Sections of the brain and spinal cord show no significant findings.

Phenomics Australia advises all research groups that images or results obtained through the services are to be acknowledged in resultant publications. Example acknowledgment: "This study utilised the Phenomics Australia Histopathology and Slide Scanning Service, University of Melbourne."

APN23/043 SVI
Macro Images
Carl Walkley

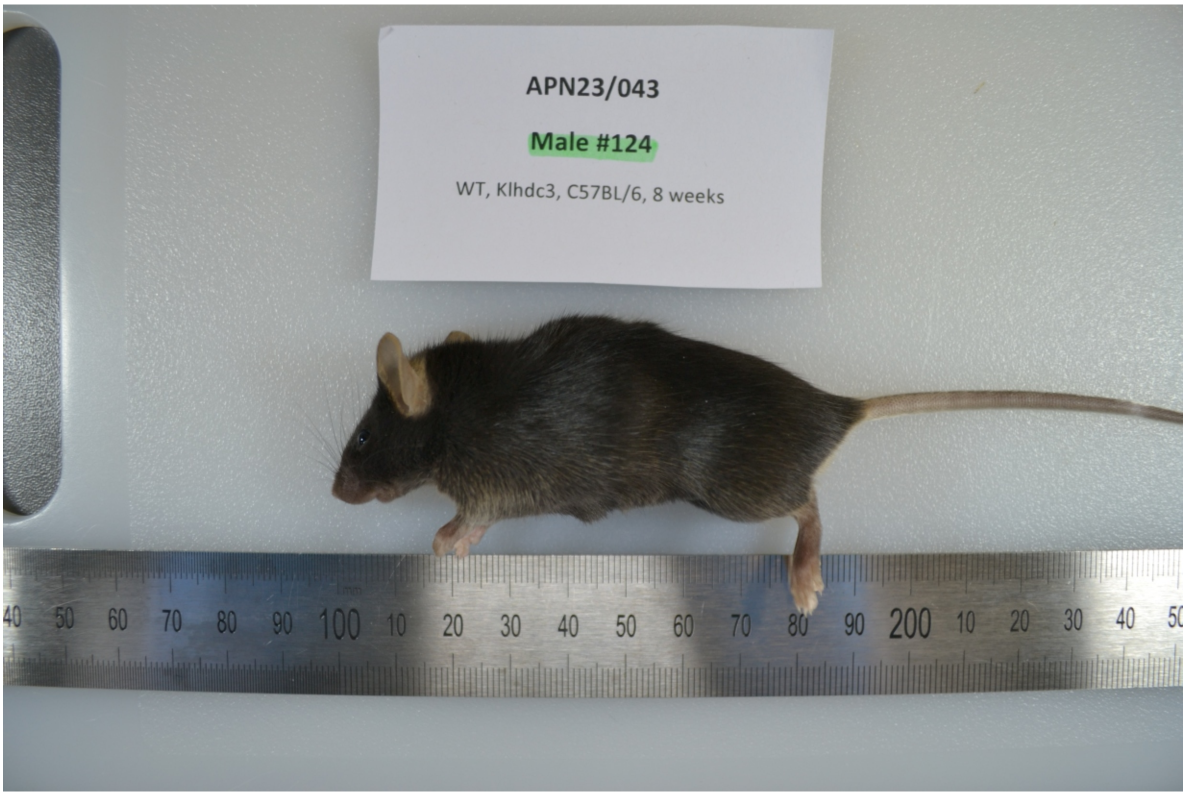

124
Klhdc3 +/+ WT Control Male

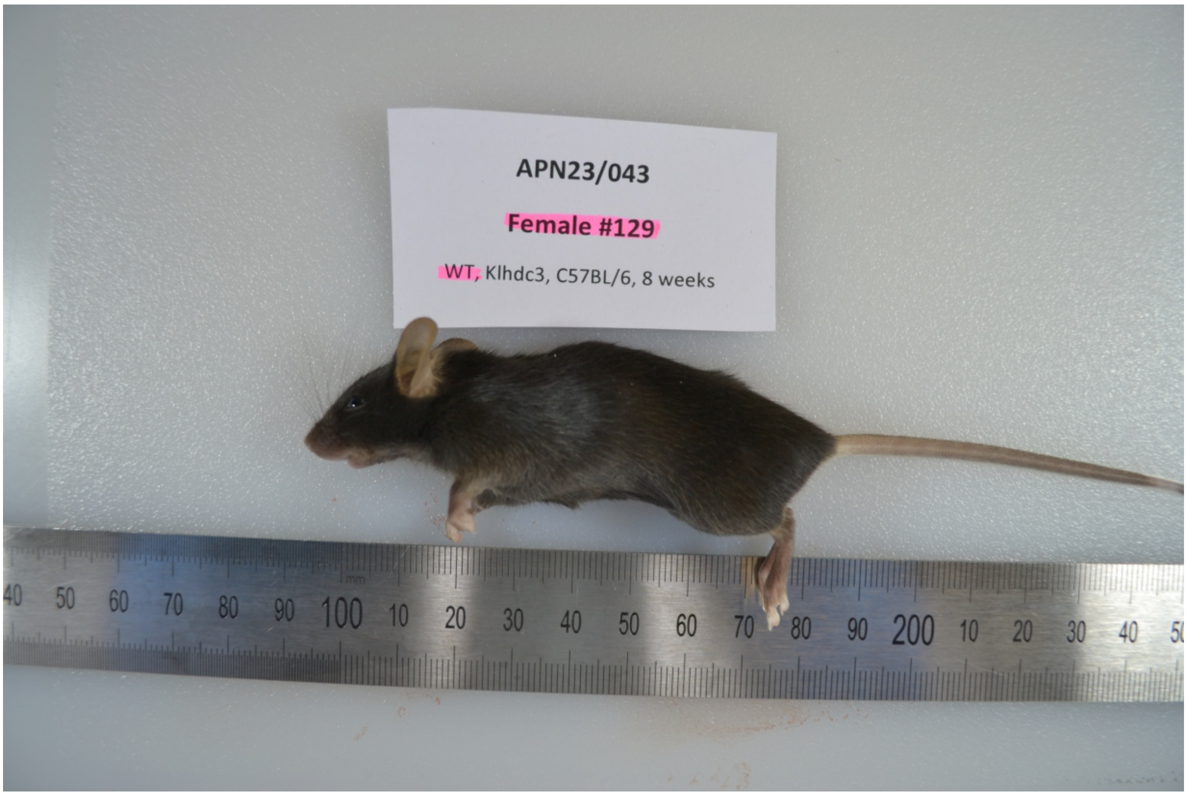

129
Klhdc3 +/+ WT Control Female

APN23/043 SVI
Macro Images
Carl Walkley

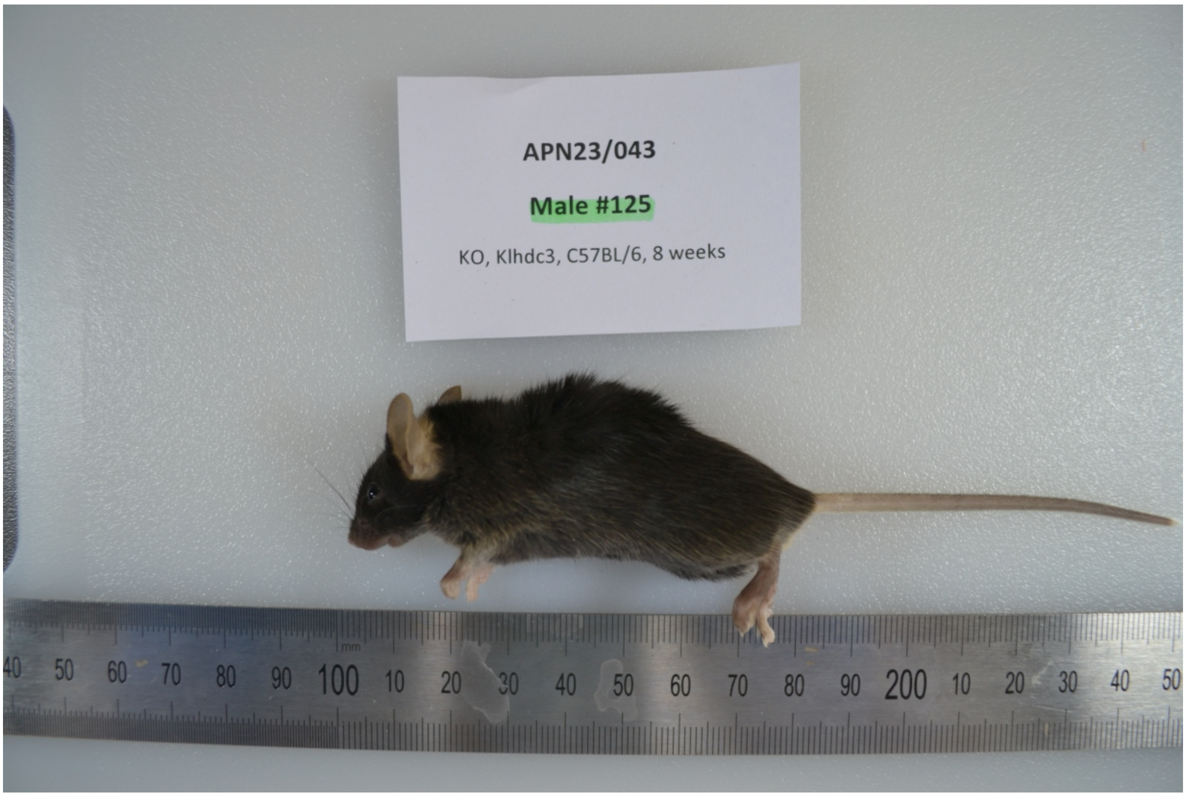

125
Klhdc3 -/- KO Male

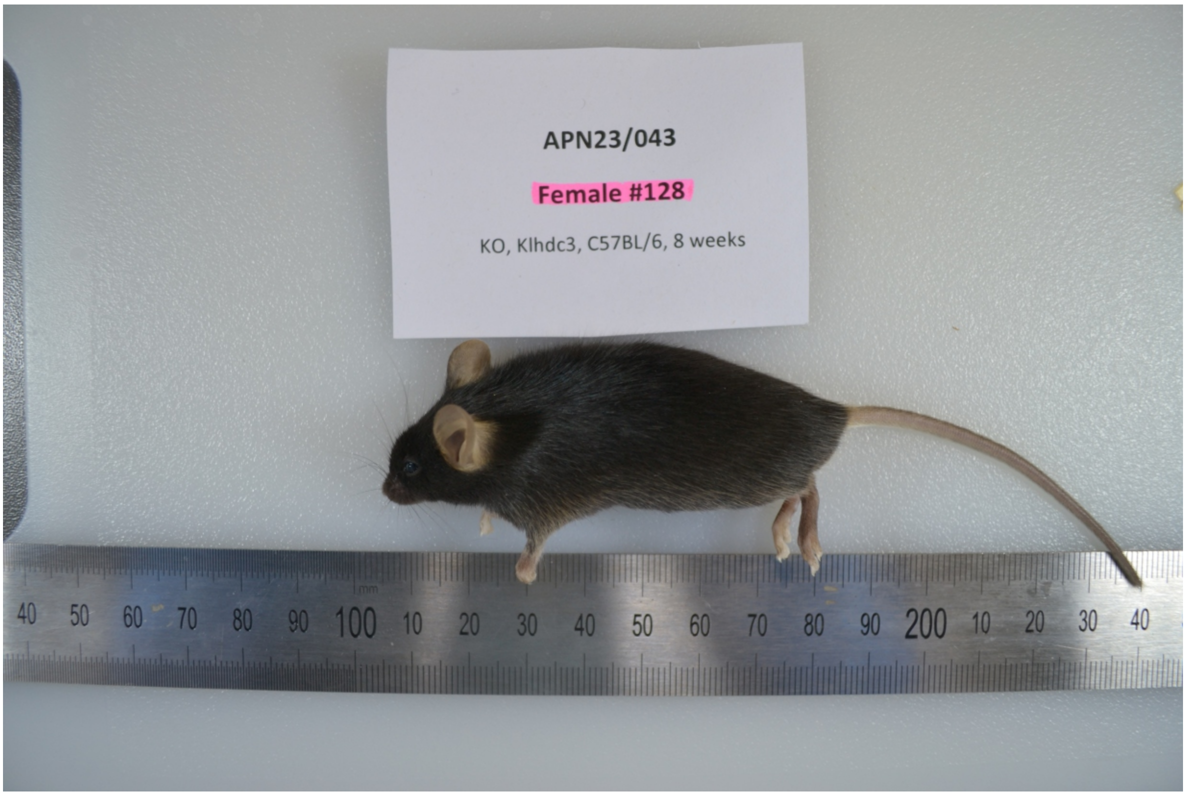

128
Klhdc3 -/- KO Female

APN23/043SVI

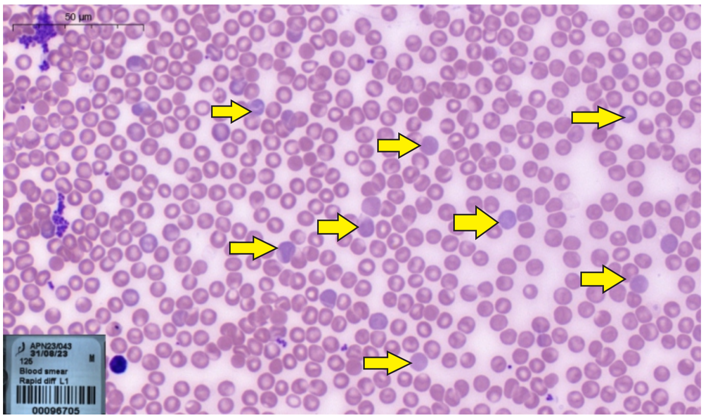

125 KO Peripheral blood smear x63
Polychromasia 96705

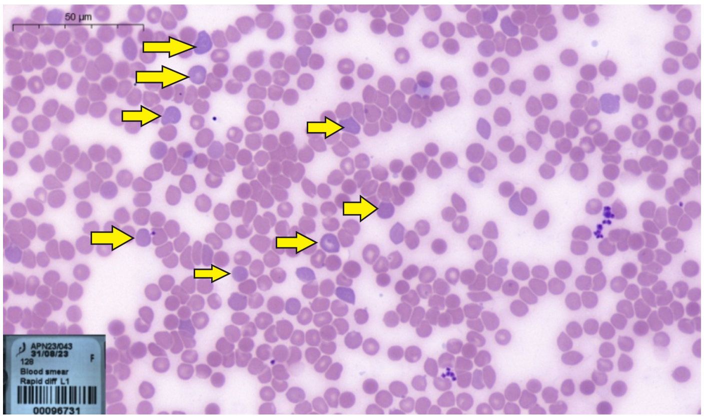

128 KO Peripheral blood smear x63
Polychromasia 96731

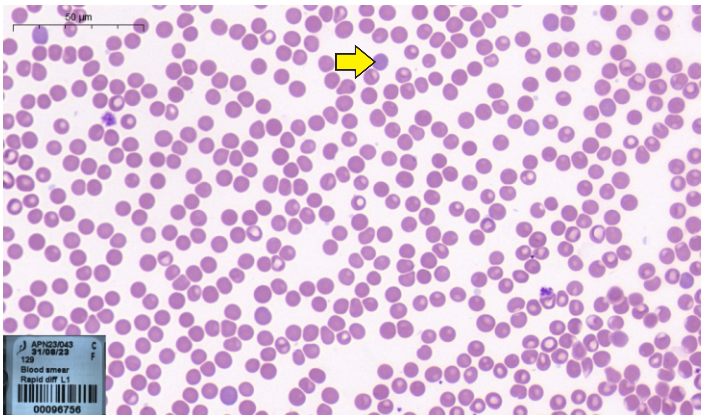

129 Peripheral blood smear x63
Scant polychromasia 96756

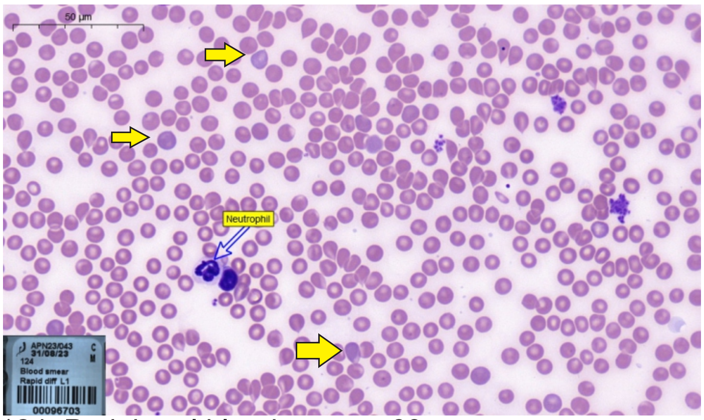

124 Peripheral blood smear x63
Scant polychromasia 96703

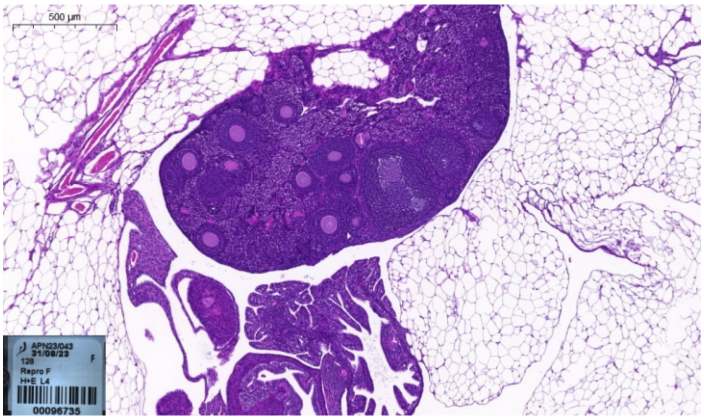

128 KO Kidney x5
Unremarkable left ovary & oviduct 96735

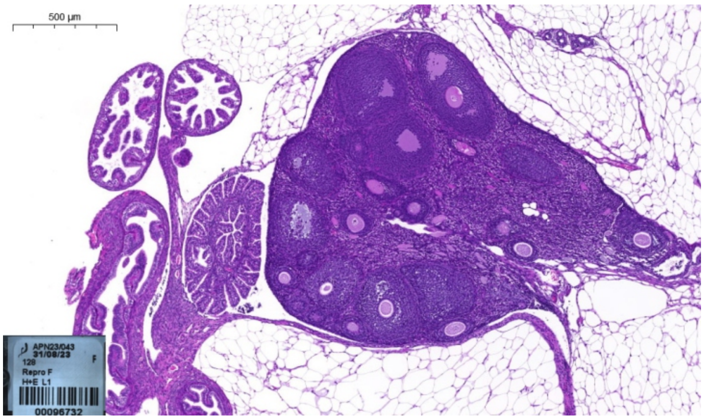

128 KO Kidney x5
Unremarkable right ovary & oviduct 96732

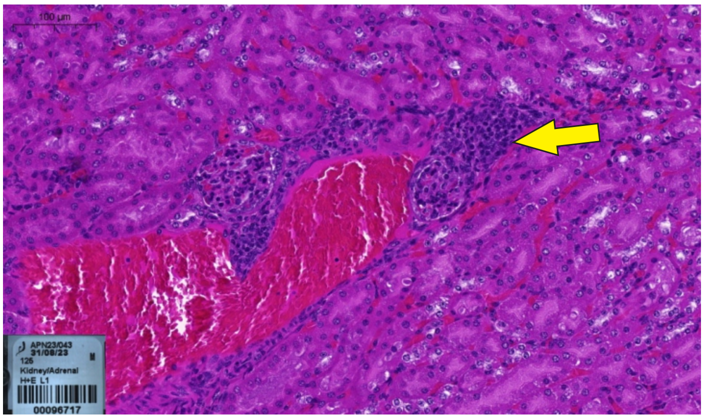

125 KO Kidney x20
Perivascular lymphocyte aggregate 96717

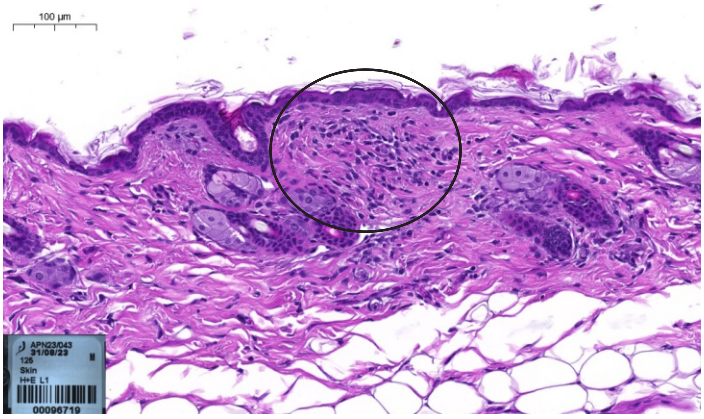

125 KO Skin x20
Lymphocyte aggregate 96719

APN23/043SVI

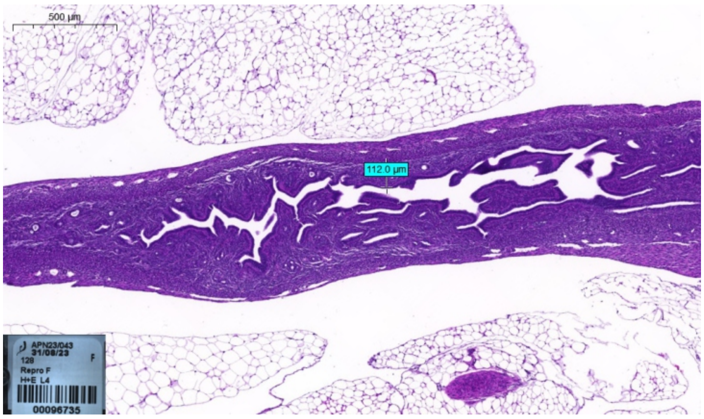

128 KO Uterus x5
Note thickness of endometrium
and number of glands 96735

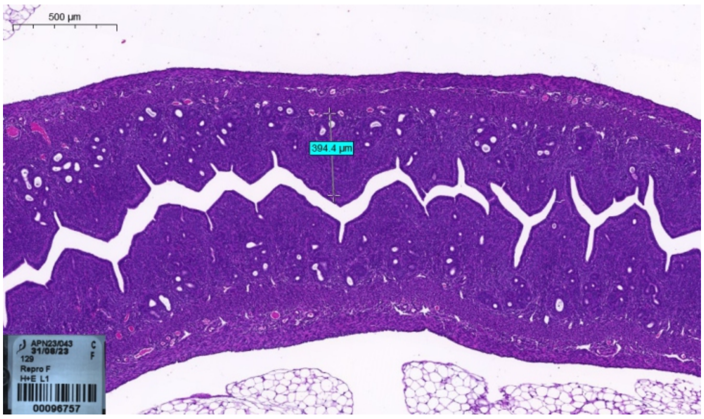

129 WT Uterus x5
Note thickness of endometrium
and number of glands 96757

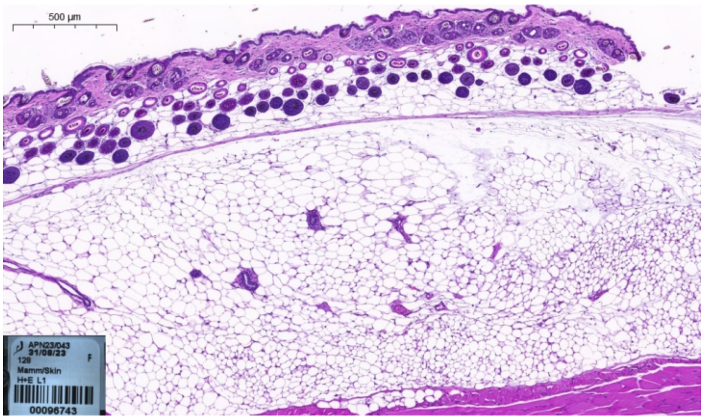

128 KO Mammary glands (inguinal) x5
Note paucity of lactiferous ducts 96743

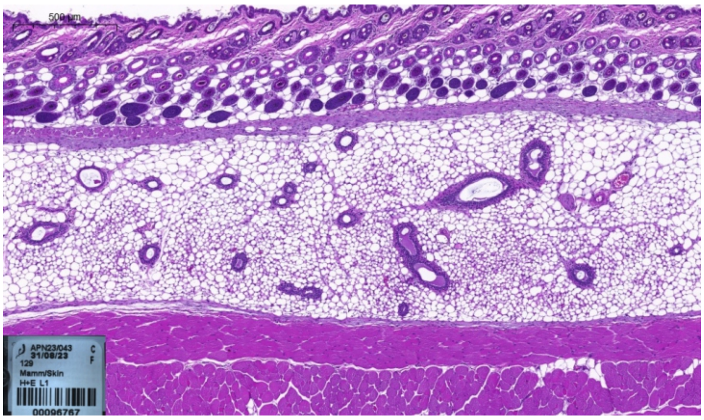

129 WT Mammary glands (inguinal) x5
Note number of lactiferous ducts 96767

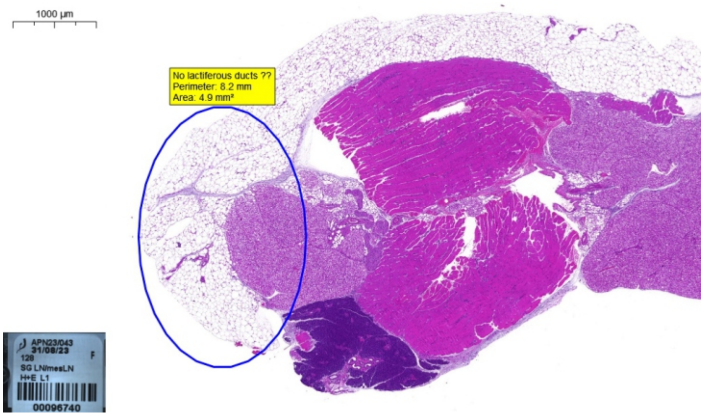

128 KO Mammary glands (cervical) x2
Note paucity of lactiferous ducts 96740

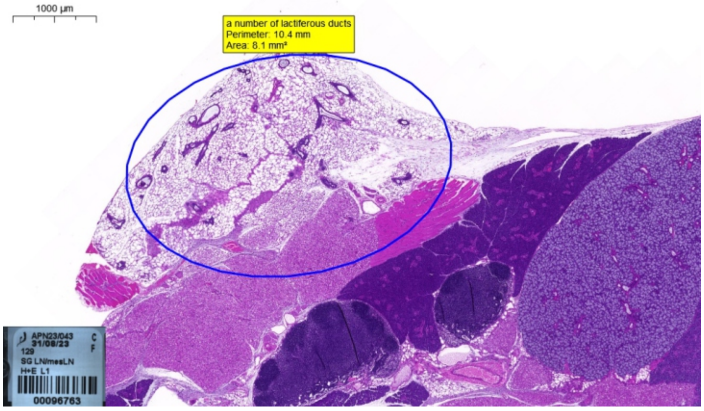

129 WT Mammary glands (cervical) x2
Note number of lactiferous ducts 96763

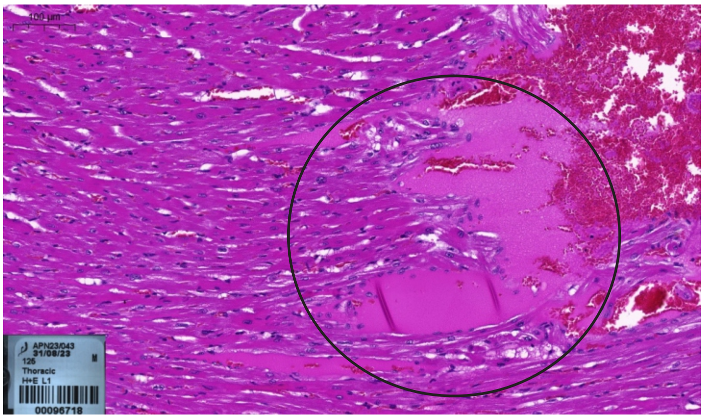
125 KO Heart x15
Cardiomyocyte vacuolation 96718
